# The Hoopoe's Uropygial Gland Hosts a Bacterial Community Influenced by the Living Conditions of the Bird

**DOI:** 10.1371/journal.pone.0139734

**Published:** 2015-10-07

**Authors:** Sonia M. Rodríguez-Ruano, Manuel Martín-Vivaldi, Antonio M. Martín-Platero, J. Pablo López-López, Juan M. Peralta-Sánchez, Magdalena Ruiz-Rodríguez, Juan J. Soler, Eva Valdivia, Manuel Martínez-Bueno

**Affiliations:** 1 Departamento de Microbiología, Universidad de Granada, Granada, Spain; 2 Departamento de Zoología, Universidad de Granada, Granada, Spain; 3 Departamento de Ecología Funcional y Evolutiva, Estación Experimental de Zonas Áridas (CSIC), Almería, Spain; Free University of Bozen/Bolzano, ITALY

## Abstract

Molecular methods have revealed that symbiotic systems involving bacteria are mostly based on whole bacterial communities. Bacterial diversity in hoopoe uropygial gland secretion is known to be mainly composed of certain strains of enterococci, but this conclusion is based solely on culture-dependent techniques. This study, by using culture-independent techniques (based on the 16S rDNA and the ribosomal intergenic spacer region) shows that the bacterial community in the uropygial gland secretion is more complex than previously thought and its composition is affected by the living conditions of the bird. Besides the known enterococci, the uropygial gland hosts other facultative anaerobic species and several obligated anaerobic species (mostly clostridia). The bacterial assemblage of this community was largely invariable among study individuals, although differences were detected between captive and wild female hoopoes, with some strains showing significantly higher prevalence in wild birds. These results alter previous views on the hoopoe-bacteria symbiosis and open a new window to further explore this system, delving into the possible sources of symbiotic bacteria (e.g. nest environments, digestive tract, winter quarters) or the possible functions of different bacterial groups in different contexts of parasitism or predation of their hoopoe host.

## Introduction

Bacteria are ubiquitous key players in a vast number of ecological and evolutionary processes [1,2]. In particular, they are frequent counterparts of symbiotic interactions, where they have been an important force shaping the evolution of a wide range of living beings, ranging from protists to all multicellular groups including plants and animals (reviewed in [3,4]). These symbiotic interactions have traditionally been classified according to their effects on host fitness as parasitism, mutualism, or commensalism. Nevertheless, distinguishing between them is not simple as they frequently have similar origins and involve similar mechanisms [5], and can change from one status to another depending on circumstances [6]. In fact, it has been demonstrated that commensal (apparently neutral) bacteria can be key in the maintenance of microbiota homeostasis and thus of host health, resembling the effects of mutualistic (clearly beneficial) symbionts [7–10]. Although parasitism draws more research efforts due to its clinical relevance (see [11,12,13] for some reviews), studies on mutualistic systems and their various known benefits for the hosts have been discovered to be a fruitful area of research. Mutualistic benefits of bacteria include processes related to nutrient uptake and assimilation, detoxification, tolerance to environmental factors and access to new ecological niches, prevention of infections and pathogen establishment, predator avoidance, signaling, and immunity development (reviewed in [4,14,15]).

Systems that involve a single bacterial strain are particularly interesting for exploring coevolutionary relationships between host and bacterial symbionts because the bacterial specificity usually implies intimate coevolutionary association. These associations have been accurately described in some systems [16–19], but the generality of these so-called two-partner symbioses has been questioned, mainly because of the detection of secondary symbionts in several of these systems [16], which implies a more relaxed coevolutionary process [17]. New data suggests that most symbiotic relationships (particularly commensalism and mutualism) involve several microorganisms, including complex microbial communities interacting with a single host, which offers a new picture of the interactions between hosts and bacteria and the multi-directional benefits involved [20].

The hoopoe (*Upupa epops*), a hole-nesting bird, has a mutualistic association with bacteria residing in its uropygial gland [21,22]. Symbiotic bacteria can produce antimicrobial substances that can benefit their host, contributing to defense against parasites and pathogens, as described in salamanders, ants and plants for example [23–25], and also in birds [26,27]. In the case of strains inhabiting hoopoe uropygial glands, several studies have shown that they provide the bird with antimicrobial substances such as bacteriocins [28] and volatile metabolites [29] that aid in protecting feathers against keratinolytic bacteria [30] and eggs against pathogens [22,31]. Until now, the hoopoe-bacteria symbiosis had been studied using culture-dependent techniques showing an association with *Enterococcus faecalis* and (less frequently) with other *Enterococcus* species [32]. However, it is well known that most microorganisms are unable to grow in laboratory conditions [33], and several lines of evidence suggest the presence of other groups of bacteria in the uropygial gland of hoopoes. For example, the elimination of bacteria from glands by means of antibiotics affected the presence of several metabolites not produced by enterococci [29], and the fingerprints of hoopoe secretions revealed several unidentified bands [34]. Additionally, microscopic studies of the glands have revealed rod-shaped bacteria and spirochetes (Martín-Vivaldi *et al*., in preparation). These evidences suggest that the microbiome hosted in hoopoe glands is a mix of strains belonging to different taxonomic groups. Therefore, the community may result from the incorporation of bacteria from different sources, such as the environment and the parental microbiota [35,36]. Indeed, we already know from cross-fostering experiments that there is an environmental influence on the enterococci strains established in nestling hoopoe glands [37]. Furthermore, the special properties of hoopoe uropygial secretions caused by bacteria in females are maintained only while incubating [21], and enterococci were not detected in the white secretions produced outside this period [22], so the symbionts (or part of them) may be acquired each breeding season. Thus, the environmental influence mediating differences in samples taken from individuals living and breeding in different conditions (i.e. captivity *vs*. wild) may be of interest to clarify the origin of the uropygial bacterial symbionts.

It has been well established that molecular techniques uncover many unculturable microbes in complex microbial samples [38–40]. Therefore, the use of molecular techniques is necessary to fully understand the relationships between hoopoes and bacteria living in their uropygial gland and the impact of the bird living conditions on its composition. This work aims to fill this gap, making a first approach to the molecular analysis and identification of the diversity of the bacterial community living in the hoopoes uropygial gland and its secretion. In addition, this study focuses on the effects of the living conditions on the community of bacteria established in the female uropygial gland, comparing wild and captive environments. The first identification of bacteria other than enterococci within the hoopoe uropygial gland secretion and the influence of bird living conditions on the composition of its bacterial community are described and discussed here.

## Materials and Methods

### Ethics statement

Research was performed in accordance with national (Real Decreto 1201/2005 de 10 de Octubre) and regional guidelines. All necessary permits to perform this research were provided by the Consejería de Medio Ambiente of the Junta de Andalucía (Spain). The study was approved by the Ethics Committee of the University of Granada (Comité de Ética en Experimentación Animal, CEEA, Ref.: 785). Suffering and stress in the birds was carefully minimized. No bird died as a consequence of its manipulation for sampling during this study.

### Study area, study species, and sampling procedures

Samples were collected during the 2006 and 2010 breeding seasons in both a wild and a captive population of hoopoes. The wild population is located in the Hoya de Guadix (southern Spain), where hoopoes breed within nest boxes placed in trees and buildings (for a more detailed description of the study area see [41,42]). The captive population was maintained at three different locations: Hoya de Guadix (Granada), the gardens of the Faculty of Science (Granada, University of Granada), and La Hoya Experimental Farm (Almería, Experimental Station for Arid Zones, CSIC). Breeding pairs were housed in independent cages (at least 3 m x 2 m x 2 m), with access to soil and provided with live food (crickets and fly larvae) and meat (beef heart) *ad libitum*. The females sampled in 2010 had been maintained in captivity at least since the previous breeding season.

During the breeding season, from mid-February to the end of July, nest boxes and cages were visited twice per week to collect uropygial gland secretion samples and record breeding parameters. All adults were ringed with numbered (Spanish Ministry of the Environment) and colored rings to aid identification. Sterile latex gloves were worn to extract uropygial gland secretions from breeding females and nestlings using a micropipette with a sterile tip introduced within the gland papilla, where secretion accumulates, after lightly washing the exterior of the gland with 96% ethanol. Samples were collected into sterile 1.5 ml microfuge tubes at 4°C and then stored at -20°C within 24 hours until processed. The extraction kit Realpure Spin Kit (Durviz S.L., Valencia, Spain) was used with 5–20 μL of each secretion sample for total nucleic acid extraction. The negative control showed no amplification for all the PCR conditions used in subsequent analyses.

### Molecular characterization of symbiotic bacterial diversity

Two different approaches were used to estimate uropygial gland bacterial diversity and to identify the most common species: (a) Ribosomal Intergenic Spacer Analysis (RISA) [43], with 36 uropygial secretions of females (23 from wild individuals and 13 from captive individuals) sampled in 2010; (b) Sequencing of the 16S rDNA and the ribosomal intergenic spacer between the rDNA 16S and rDNA 23S genes. Partial 16S sequences were obtained from a Temporal Temperature Gradient Electrophoresis (TTGE) and ribosomal intergenic spacers from RISA profiles. The 11 most prevalent bands from TTGEs and the five most prevalent bands from RISA fingerprints were selected for sequencing. In addition, we built clone libraries for the 16S rDNA and for the ribosomal intergenic spacer region. Clone libraries of the 16S rDNA were obtained from three samples (two from 2010 and one from 2006) and clone libraries of the ribosomal intergenic spacer from nine samples (all from 2010).

#### Fingerprinting of bacterial communities

The RISA PCR from genomic DNA was performed according to [44] using the oligonucleotide primers 72f (5’-TGC GGC TGG ATC TCC TT–3’) and 38r (5’-CCG GGT TTC CCC ATT CGG–3’) [44,45]. TTGE samples were prepared by nested PCR using the oligonucleotide primers W01 (5′-AGA GTT TGA TC[AC] TGG CTC–3′) and W012 (5′-TAC GCA TTT CAC C[GT]C TACA–3′) in the first PCR, and HDA1-GC (5’-CGC CCG GGG CGC GCC CCG GGC GGG GCG GGG GCA CGG GGG GAC TCC TAC GGG AGG CAG CAGT–3’) and HDA2 (5’- GTA TTA CCG CGG CTG CTG GCA–3’) in the second PCR. TTGE PCR and electrophoresis were performed following [46]. The 50 μL PCR reactions for both RISA and TTGE were carried out in a Mastercycler thermocycler (Eppendorf, Hamburg, Germany) using a MBL-*Taq* DNA polymerase (Dominion-MBL, Cordoba, Spain). Gels were stained in ethidium bromide and photographed under UV transillumination using a UVP ImageStore 5000 system (Ultraviolet Products, Cambridge, UK).

After running the polyacrylamide gels to separate the bands produced by the amplification of the ribosomal intergenic spacer, the resulting images were processed with the Fingerprinting II Informatix Software (Bio-Rad, Hercules, California, USA) to standardize them and make them comparable. A dendrogram was then constructed using the Unweighted Pair Group Method with Arithmetic averages (UPGMA) algorithm based on the Jaccard similarity coefficients using the same software. We also compared groups of samples determined based on their living conditions (captive *vs*. wild), using a non-parametric permutation MANOVA test (Adonis function) included in the Community Ecology Package Vegan [47] for the R statistical software [48]. The richness was considered as the number of observed OTUs (Operational Taxonomic Units) recognized as different bands in our fingerprints. Beta diversity analyses to compare community composition between samples (i.e. Principal Coordinate Analysis (PCoA) based on the Jaccard similarity matrix) were performed using scripts from the Quantitative Insights Into Microbial Ecology (QIIME, version 1.8) pipeline [49] and the EMPeror software for graphic representations [50]. All other statistical analyses (including Mann-Whitney U-tests and Kruskal-Wallis tests for diversity comparisons and the two-sample test for equality of proportions with continuity correction to check the relative abundance of OTUs within different groups) were performed using the respective functions within the R Stats Package included in the R statistical software [48]. A Kruskal-Wallis test was performed to ensure that the three populations maintained in captivity could be used as one captive population in subsequent analyses (Kruskal-Wallis tests, N = 13; χ^2^ = 0.94; P = 0.62; Almería, N = 8, samples mean (SD) = 15.0 (3.1); Faculty of Science, N = 4, samples mean (SD) = 16.3 (0.8); Guadix, N = 1, samples mean (SD) = 15.0 (0.0)). The taxonomic assignment of specific bands in our samples based on the sequencing of the five most prevalent bands in the fingerprint patterns was performed using the band-matching option in the Fingerprinting II Informatix Software (Bio-Rad, Hercules, California, USA), with 1% position tolerance and 0% optimization. The 36 analyzed samples from 2010 females were divided into two groups based on their living conditions (maintained in captivity, N = 13, and wild, N = 23). Both groups contained a single sample per individual to avoid pseudoreplication. The consistency of the groups regarding sample origin was analyzed using resampling based on the Jackknife average method [51].

#### Clone libraries, sequencing, and OTU identification

A fragment of 700 base pairs (bp) from the 16S rDNA (including V1–V4 regions) from three samples and the variable ribosomal intergenic spacer from bacteria in the secretions of nine samples was amplified as described above. Then the PCR products for each region were pooled. The mixed PCR products were cloned on pGEM-T Easy Vector according to the manufacturer's instructions (Promega Corporation, Madison, Wisconsin, USA) to generate clone libraries for the 16S rDNA and the ribosomal intergenic spacer.

The 29 selected clones for the 16S rDNA and the 21 for the ribosomal intergenic spacer libraries were characterized by TTGE and RISA respectively to group them by similarity in order to sequence only one per group. The result was 12 different groups for the 16S rDNA and 13 groups for the ribosomal intergenic spacer. One clone from each group was then used for sequencing from plasmid DNA preparations using an ABI Prism 310 Genetic Analyzer (Applied Biosystems, Life Technologies, Madrid, Spain). Additionally, the fragments obtained from the bands isolated from TTGE and RISA fingerprints were also sequenced. The bands selected for sequencing were purified from the polyacrylamide gels using passive diffusion in sterilized water (12 h at 4°C), and were then re-amplified using the same primer and reaction conditions as before to be sequenced.

Before identification, the sequences obtained for the same marker (either 16S rDNA or ribosomal intergenic spacer) from clones and fingerprint bands were compared by aligning them with Clustal 2.1 [52]. Sequences with 100% identity were considered the same, and only one (the longest) was used for the systematic study. A total of 18 different sequences have been deposited in the NCBI GenBank database with accession numbers KR076707 to KR076724.

The MegaBLAST module of the BLAST (Basic Local Alignment Search Tool) Sequence Analysis Tool BLASTN 2.2.30+ (available online at the National Center for Biotechnology Information (NCBI) website (Bethesda, USA, http://www.ncbi.nlm.nih.gov/)) was used to search for the closest phylogenetic neighbors using local alignments of our sequences with those in the GenBank Nucleotide collection (nr/nt) database. The overall phylogenetic similarity was evaluated using an expected threshold of 10 and all the default parameters for MegaBLAST. For each OTU sequence (i.e. the different sequences retained), we selected the five different known strains with the highest Max Score, and also included all known species in the list whose Query Cover or Identity percentage were within the range of those of the five selected species. By following these criteria, we ensured that all the most probable candidates closely related to our OTUs were used for phylogenetic analyses. Whenever possible, two different strains per candidate species were included for each OTU. After this step, we detected that our ribosomal intergenic spacer candidates (due to the high variability in this region) did not have homology with the database sequences throughout all their length, but only in two clearly differentiated subregions (presumably associated to two tRNA genes, for alanine and isoleucine, that have been found in the ribosomal intergenic spacer amplified in other studies [53–55]). Since these two subregions were not present simultaneously (nor were either of them present in all the sequences), they were used separately to avoid errors in the grouping of candidates with different subregions. Moreover, eight of the candidate sequences for the ribosomal intergenic spacer did not correctly align with the subregions used, and were therefore excluded from further analyses.

Sequences from these species were included together with those of our OTUs to build three different consensus trees (one for the 16S rDNA and another two for the two homology subregions within the ribosomal intergenic spacer), where our OTUs could be taxonomically situated. The sequences were aligned with Clustal 2.1 [52] using the default parameters, including one outgroup sequence for the pertinent region in each case from the algae *Caulerpa taxifolia*. Then a standard bootstrap was performed with the Seqboot tool included in Phylip 3.695 [56] and 100 trees were built corresponding to each subsample with FastTree 2.1.3 [57,58]. Finally, a consensus tree was built using the Consense tool included in Phylip 3.695 [56]. The bootstrapping values were incorporated with the Compare-To-Bootstrap script from the MicrobesOnline tree comparison tools (available at http://www.microbesonline.org/fasttree/treecmp.html). Image editing of the trees was performed using FigTree 1.4.1 [59].

## Results

### Bacterial diversity in hoopoe uropygial glands

**Fig 1:**
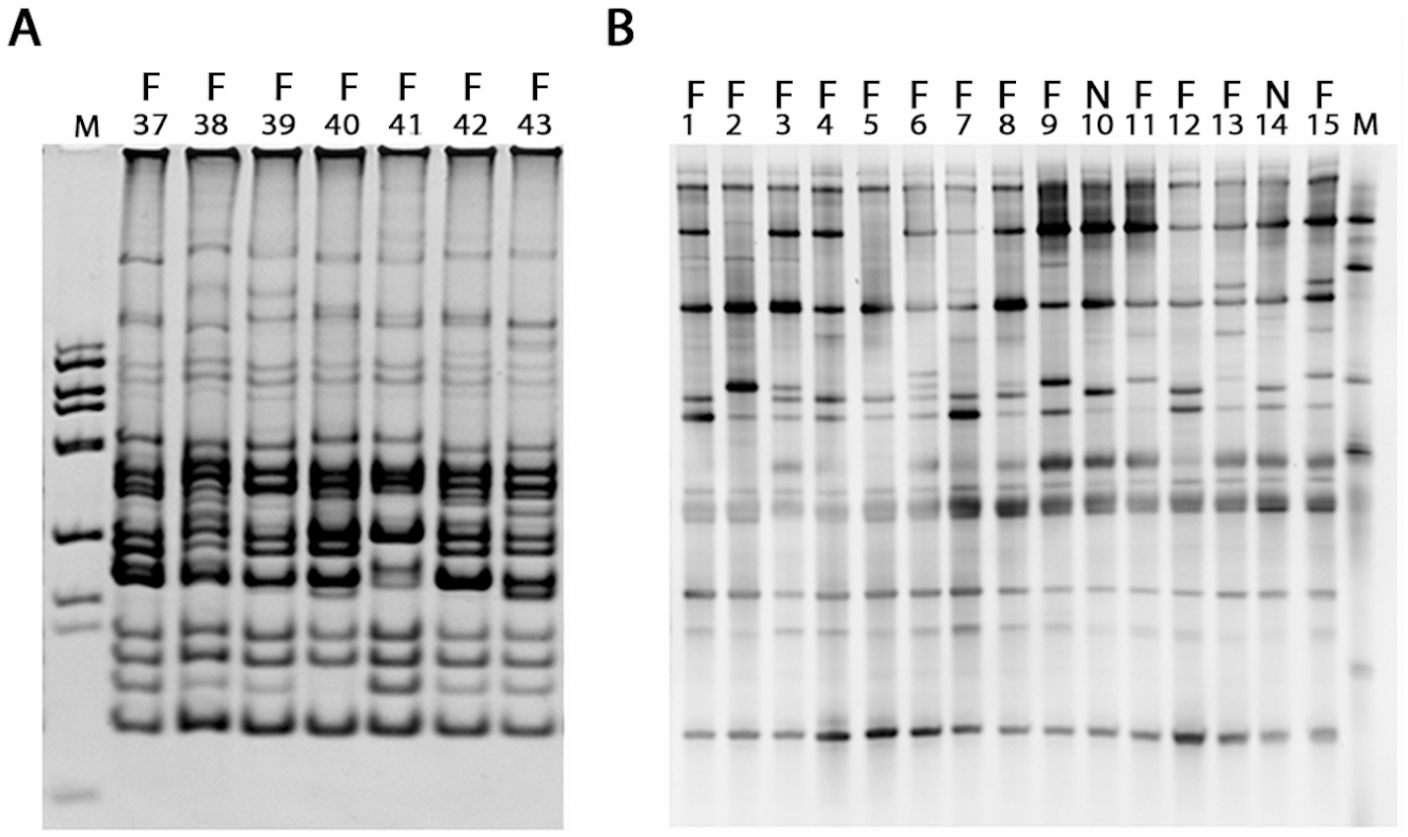
Uropygial bacterial community fingerprints. (A) Example of RISA gel with the band profiles of uropygial secretions of seven different wild hoopoe females used in this study. Lane M contained a 100-bp DNA ladder (Biotools, B&M Labs, Madrid, Spain). (B) TTGE gel showing the characteristic band profiles of the uropygial secretions of 13 wild hoopoe females (F), and also of two wild nestlings (N) to show their profile resemblance with those of females; lane M contained a band profile of bacterial strains from the laboratory collection.

The analyses of RISA and TTGE fingerprints revealed that the bacterial community inhabiting hoopoe uropygial glands is more complex than previously thought, showing 11 to 21 different bands per individual (Fig 1). Nevertheless, the community was rather uniform among hoopoes, with 24% of the bands detected in RISA gels (N = 46) present in at least 50% of individuals (Fig 1A). The fingerprint profiles of nestlings looked very similar to those of females (Fig 1B). However, given the low number of nestlings sampled they were not used in our statistical comparison.

### Differences between wild and captive hoopoes

**Fig 2:**
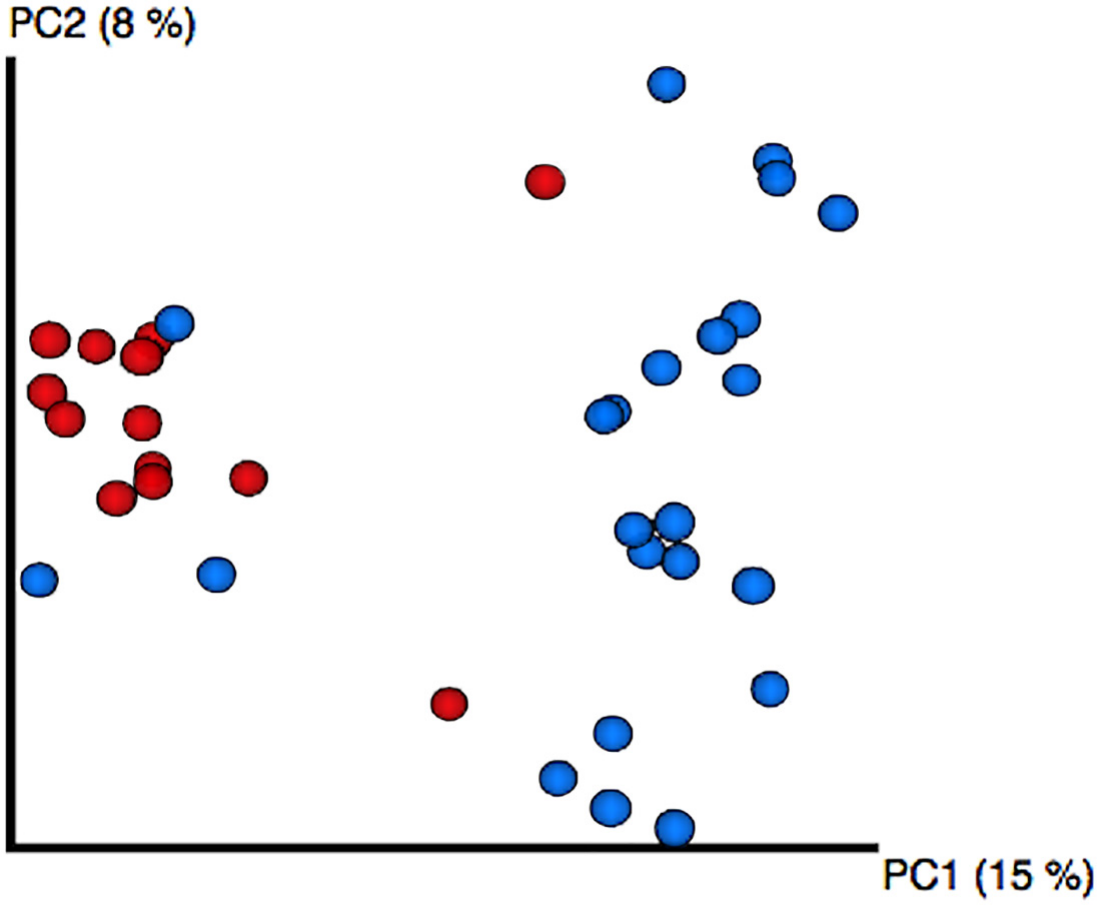
PCoA analysis of captive and wild hoopoe female samples. The PCoA shows sample grouping by similarities in OTU composition. Samples from captive (red) and wild (blue) female hoopoes cluster separately. The percentage of variation explained by the plotted principal components (PC) is indicated in parentheses.

We compared the bacterial communities reflected in RISA gels among populations, and found that the richness (i.e. number of bands) in wild samples was non-significantly higher than in captive samples (Mann-Whitney U-test, N = 36; U = 93.5; P = 0.062; wild samples mean (SD) = 17.0 (2.2), captive samples mean (SD) = 15.4 (2.6)). When taking into account the identity of the OTUs in the communities, bacterial communities from females maintained in captivity differed significantly from those from wild females (Adonis, F_1,35_ = 16.89; P < 0.001; Fig 2). The consistency of the groups based on sample origin showed a high percentage (above 84%) of correct assignments for both captive and wild samples to their corresponding group when resampling our dataset. In addition, the Principal Coordinate Analysis (PCoA) confirmed our results, as both wild and captive female samples clustered separately. According to the PCoA, the difference between these populations explained 15% of the variation among bacterial communities, with both principal components (PC) 1 and 2 accumulating 23% of total variation in our samples.

### Species composition

**Fig 3:**
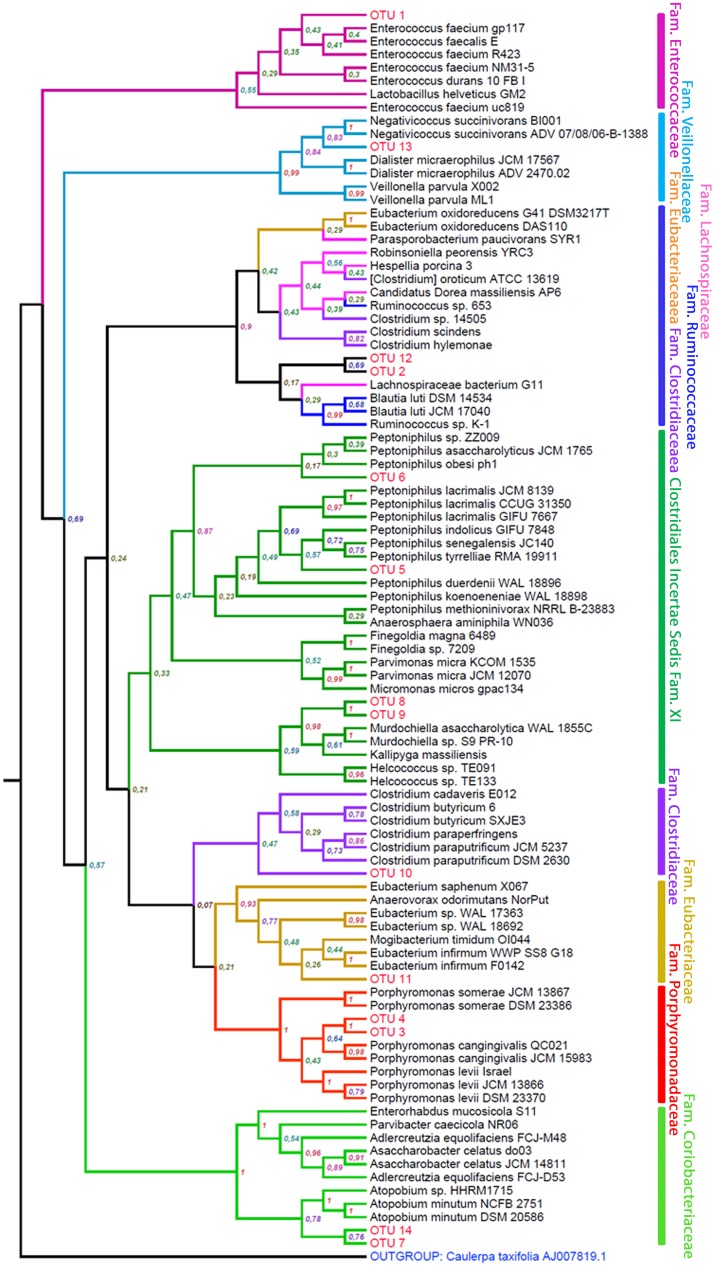
Consensus tree with the taxonomic position of the different bacterial OTUs detected in hoopoe uropygial secretions by sequencing the 16S rDNA. Labels in nodes indicate the bootstrap mean probability for each clade after 100 repetitions.

The 16S sequences revealed 14 different OTUs (i.e. the different sequences identified; Fig 3), most (10 out of 14) grouped within clades of the phylum *Firmicutes*, two within the phylum *Actinobacteria* (family *Coriobacteriaceae*), and the remaining two within the phylum *Bacteroidetes* (genus *Porphyromonas*) (Fig 3, Table 1). Among the *Firmicutes*, OTU 1 was an *Enterococcus* species and probably belongs to *E*. *faecium* or *E*. *faecalis* (Fig 3). We found two different *Peptoniphilus* species (OTU 5 and 6), and within the same family (*Clostridiales Incertae Sedis XI*), two strains close to the genera *Murdochiella* and *Kallipyga* (OTUs 8 and 9). OTU 10 grouped with the genus *Clostridium* but did not match any of the candidate species. The three remaining OTUs grouped in the order *Clostridiales* were an *Eubacteriaceae* (OTU 11) close to several species of the genus *Eubacterium* and two species (OTUs 2 and 12) linked to a clade of *Ruminococcaceae* (but with low consistency; Fig 3). In four cases (8 OTUs), two of our OTUs were paired in the tree, with different bootstrap probabilities. In two instances (OTUs 3+4 and 8+9), that probability was 100% (Fig 3). These OTUs were therefore considered as strains of the same species since, in all the other cases of 100% probability in the grouping of two sequences, the taxa were two strains of the same species (Fig 3, 10 cases).

**Table 1:** Taxonomic position of the OTUs detected in the uropygial secretion of female and nestling hoopoes.

TAXONOMY						OTUs	
Phylum	Class	Order (Suborder)	Family	Genus	Species	16S	ITS
*Firmicutes*	*Clostridia*	*Clostridiales*	*Incertae Sedis XI*	*Peptoniphilus*	*Peptoniphilus* sp1	OTU 5	
					*Peptoniphilus* sp2	OTU 6	
					*Peptoniphilus* sp3		ITS 8
				*(Murdochiella/ Kallipyga)*	Sp1 strain 1	OTU 8	
					Sp1 strain 2	OTU 9	
			*Eubacteriaceae*	*Eubacterium*	*Eubacterium* sp	OTU 11	
			*Lachnospiraceae*	*(Coprococcus)*	Sp		ITS 5
			*Clostridiaceae*	*(Clostridium)*	Sp	OTU 10	
				*Clostridium*	*Clostridium* sp		ITS 6
			Unknown	Unknown	Sp1	OTU 2	
					Sp2	OTU 12	
		*Thermoanaerobacteriales*	*Thermoanaerobacteriaceae*	*(Thermoanaerobacterium)*	Sp		ITS 1
	*Bacilli*	*Lactobacillales*	*Enterococcaceae*	*Enterococcus*	*Enterococcus* sp	OTU 1	ITS 2
		*Bacillales*	*Paenibacillaceae*	*(Paenibacillus)*	Sp		ITS 3
	*Negativicutes*	*Selenomonadales*	*Veillonellaceae*	*(Negativicoccus)*	Sp	OTU 13	
				Unknown	Sp		ITS 4
*Actinobacteria*	*Actinobacteria*	*Actinomycetales*	Unknown	Unknown	Sp		ITS 7
	*Coriobacteria*	*Coriobacteriales* (*Coriobacterineae*)	*Coriobacteriaceae*	*(Atopobium)*	Sp1	OTU 7	
					Sp2	OTU 14	
*Bacteroidetes*	*Bacteroidia*	*Bacteroidales*	*Porphyromonadaceae*	*Porphyromonas*	*Porphyromonas* sp1 strain 1	OTU 3	
					*Porphyromonas* sp1 strain 2	OTU 4	

**Fig 4:**
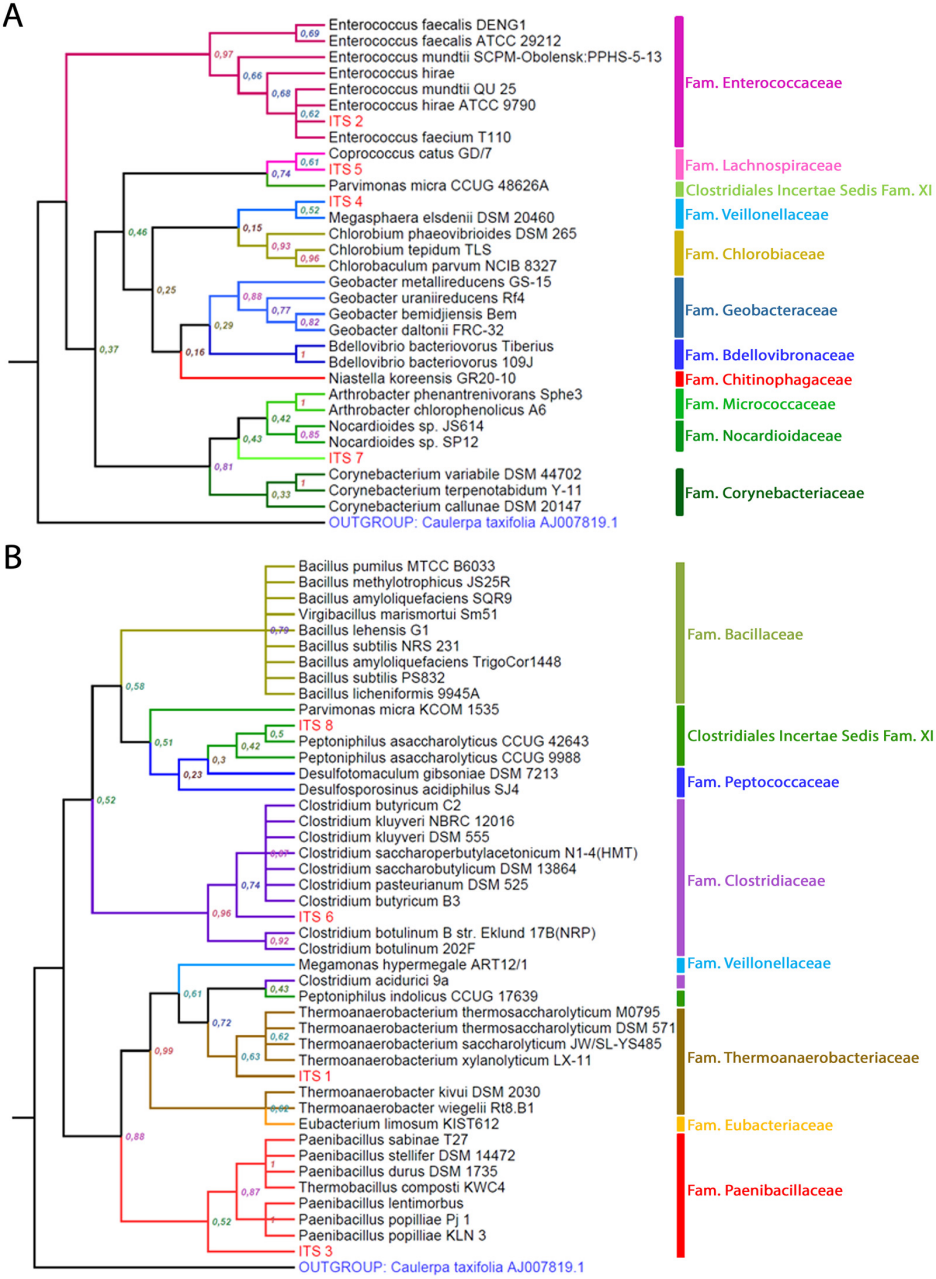
Consensus trees with the taxonomic position of the different bacterial OTUs (ITS) detected in hoopoe uropygial secretions by sequencing the ribosomal intergenic spacer. Panels A and B include OTUs grouped according to each of the two subregions found within the ribosomal intergenic spacer. Labels in nodes indicate the bootstrap mean probability for each clade after 100 repetitions.

Taxonomic position inferred for the OTUs detected in the uropygial secretion of female and nestling hoopoes by several different molecular methods. When the sequences were not clearly included within a genus in the trees, the genus most closely related to the sequence is given in parentheses (see Figs 3 and 4).

The analysis of ribosomal intergenic spacer sequences revealed eight different OTUs (in this case called ITS; Fig 4), most included within the phylum *Firmicutes* (7 out of 8), and only one (ITS 7) grouped with the phylum *Actinobacteria* (order *Actinomycetales*) (Table 1). In the *Firmicutes*, two OTUs could be clearly assigned to the genus level. ITS 2 grouped with *Enterococcus* species and ITS 6 grouped with *Clostridium* species. These two OTUs, together with ITS 8 (grouped within *Clostridiales Incertae Sedis XI* in the genus *Peptoniphilus*), coincided with taxa detected using the 16S marker (Table 1). However, the ribosomal intergenic spacer marker provided the identification of five additional OTUs in groups not detected among the 16S sequences. ITS 1 was included in a clade within the family *Thermoanaerobacteriaceae* (close to the *Thermoanaerobacterium* species), ITS 4 formed a clade with a *Megasphaera* species (family *Veillonellaceae*), ITS 5 was grouped with a *Coprococcus* species (family *Lachnospiraceae*), and ITS 3 was grouped close to the family *Paenibacillaceae*. The ribosomal intergenic spacer approach, as the use of 16S (Table 1), identified an OTU within the *Actinobacteria* phylum (ITS 7), although in this case it was not a *Coriobacteriaceae* but was related to the families *Nocardioidaceae* and *Micrococcaceae*.

In no case did one of our OTUs (either from 16S or from the ribosomal intergenic spacer) get paired with a known species with a very high probability (more than 90%), so at most we could assign them to particular genera or families according to their position in the consensus trees (Table 1). OTUs were assigned to the family of the known species (from the GenBank Nucleotide collection database) they were more closely related to in the consensus trees. OTUs were assigned to a particular genus only if their sequence resulted clearly included within a clade of different species of the same genus. When an OTU was not included in a particular genus but grouped with one, it was considered to be related to that genus (in parentheses in Table 1).

### Composition of wild and captive hoopoes uropygial microbiomes

The comparison between captive and wild hoopoes in the frequency of appearance of the five most prevalent bands in RISA fingerprints revealed some differences (Table 2). There were two OTUs that appeared in most samples of both populations, while in the other three cases prevalence differed between them. ITS 8 (*Peptoniphilus* species) was present in 100% of the analyzed samples (N = 36). ITS 7 (an *Actinobacteria* species close to *Nocardioidaceae* and *Micrococcaceae*) also appeared in a high percentage of samples regardless of their origin; in fact, it occurred in a similar percentage in both sample groups, as confirmed by a two-sample test for equality of proportions with continuity correction (N = 36; χ^2^ = 0.11; P = 0.736). In the other three cases, prevalence was higher in samples from wild females than in samples from captive females (Table 2), although differences were significant only for ITS 4 (probably a *Veillonellaceae* species, N = 36; χ^2^ = 15.36; P < 0.001) and ITS 6 (a *Clostridium* species, N = 36; χ^2^ = 6.40; P = 0. 011), whereas ITS 5 (probably a *Lachnospiraceae*) was more common in wild females but not significantly so (N = 36; χ^2^ = 1.97; P = 0.161).

**Table 2:** Frequency of appearance of ITS OTUs in wild and captive hoopoe females.

Phylum	Class	Order	Family	Genus	OTU	% wild females (N = 23)	% captive females (N = 13)
*Firmicutes*	*Clostridia*	*Clostridiales*	*Lachnospiraceae*	*-*	ITS 5	34.8	7.7
			*Clostridiaceae*	*Clostridium*	ITS 6	65.2	15.4
			*Incertae Sedis XI*	*Peptoniphilus*	ITS 8	100.0	100.0
	*Negativicutes*	*Selemonadales*	*Veillonellaceae*	*-*	ITS 4	73.9	0.0
*Actinobacteria*	*Actinobacteria*	*Actinomycetales*	-	*-*	ITS 7	91.3	100.0

Comparison of the frequency of appearance of the five most prevalent bands (sequenced from RISA gels) between the uropygial secretions of wild hoopoe females and females maintained in captivity.

## Discussion

Molecular analysis of the bacterial community in hoopoe uropygial secretions revealed that it is more complex than previously found by culture-dependent methods. This study shows that, apart from *Enterococcus* species (the predominant bacteria found in hoopoe glands to date [22,60]), the dark secretions of breeding females and nestlings host at least 20 different OTUs from three different bacterial phyla (*Firmicutes*, *Actinobacteria*, and *Bacteroidetes*). The communities were very similar among individuals, indicating a specific, largely invariable assemblage of bacterial species residing in this particular bird organ, although a deeper sampling (for example using high-throughput sequencing) may be needed to confirm this finding. Interestingly, the bacterial OTUs in hoopoe glands have rarely resulted in the identification of known species, suggesting that this environment has a high potential to uncover strains with interesting properties.

This is the first time that bacteria other than enterococci are found to be abundant within the uropygial gland of birds. Multipartite mutualistic symbioses of animals with microorganisms are the norm rather than the exception (reviewed in [20]). The stability of this kind of associations requires that different symbionts provide independent benefits to hosts or that, if producing redundant benefits, counterbalance their relative abundances or coevolve to shared pathways of resource consumption and production [20]. It is worth noting that the bacteria in hoopoe glands belong to a wide taxonomic range (at least 18 different species corresponding to 16 different genera in 13 families). We have previously shown that, within the *Enterococcus* genus, species and strains with higher antimicrobial potential (mediated by bacteriocin production) prevail in hoopoe uropygial secretions [32]. Although hoopoes may regulate the gland colonization by bacteria to keep an adequate assembly, these results suggest that the competition ability of different strains colonizing the uropygial gland of hoopoes determines the final bacterial communities [61]. The coexistence in the gland of several unrelated species from different families opens the possibility of unrelated bacterial strains providing complementary benefits to hosts. The main benefit demonstrated for enterococci inhabiting the gland is the production of bacteriocins defending hoopoes from feather-degrading bacteria [30] and eggs from trans-shell infection of embryos [22,31]. Other species of the bacterial community in the uropygial secretion may be responsible for certain chemicals detected in the secretion that increase its antimicrobial potential. Indeed, we have shown that the experimental eradication of bacteria from glands by antibiotics eliminates several volatile compounds with antimicrobial activity, some of them typical metabolites from various of the bacterial groups detected in this study (such as 4-methyl pentanoic and 3-phenyl propanoic acids produced by clostridia) [29]. It is well known that *Actinobacteria* are especially able to produce protective substances and are involved in mutualisms with animals that benefit from their antifungal capabilities [62]. One *Actinobacteria* genus related to the *Actinomycetales* found in hoopoe glands (*Nocardioides*) has been confirmed as an antibiotic-producing mutualist in fungus-growing ants [63]. *Atopobium*, a group close to *Coriobacterium* [64], is involved in nutritional endosymbiosis with *Pyrrhocorix* bugs [65]. Therefore, these bacteria are good candidates to act as beneficial strains in hoopoes as well. Additionally, some of the bacteria detected in the gland of hoopoes are likely responsible for the stench of the secretion. This may confer protection against predators [66], as occurs for the closely related green woodhoopoe [67]. Future studies should examine the specific role in secretion functionality of the different bacterium species integrating the bacterial community.

All the genera discovered within the bacterial community of the hoopoe uropygial gland are obligated or facultative anaerobes. This result was expected since the gland ampulla where the secretion accumulates is near a sphincter that isolates the cavity from the surrounding air, and the viscosity of the secretion itself may prevent oxygen diffusion. Although hosts would benefit from antimicrobial compounds produced within the uropygial gland, only aerotolerant antibiotic-producing bacteria such as enterococci would be able to grow directly on host tissues (feathers and eggshells). However, strictly anaerobic bacteria may be able to grow in the numerous crypts of hoopoe eggshells, which rapidly fill with bacteria-rich secretion during incubation, thereby providing the anaerobic environment required by some of the bacteria detected [31]. Further research is needed on the composition of communities established on eggshells to confirm these hypotheses.

Our results emphasize the importance of molecular methods to characterize the symbiotic community of the hoopoe uropygial gland. Nevertheless, even though molecular methods are powerful tools for the study of bacterial communities [68], results may vary depending on the method employed. In this study, we used two different approaches (sequencing of bands from fingerprints and clone libraries) for two different markers (16S rDNA and ribosomal intergenic spacer), which produced complementary results. Remarkably, enterococci (the taxonomic group whose presence and importance in this system has been repeatedly confirmed by culture-dependent methods [22,30–32,60]) have only been detected by sequencing of bands from fingerprints and not in clone libraries. This disparity is probably because this group is in low abundance in comparison to other bacteria groups within the uropygial secretion. The combination of methods used here suggests that we have detected the most prevalent (selected in the fingerprints) and abundant (cloned in libraries) strains residing in hoopoe uropygial gland secretions. Nevertheless, in future research on this topic other approaches such as high-throughput sequencing may be needed to describe the whole uropygial bacterial diversity, as well as the relative abundances of the different strains.

The diversity of bacterial communities in terms of composition differed for captive and wild hoopoes, suggesting environmental influences on the uropygial bacterial community assemblage. Although the variation among our bacterial populations explained by the first two axes of the PCoA constituted only 23% of the total, this is a common result in diversity studies (see for example [69,70,71]). Most of the variation among bacterial communities comes from rare strains that are less relevant than predominant ones when describing such communities, and whose influence is usually not included within the first PCoA axes [72]. Environmental influences have previously been described for the enterococci population [37], and our own results now extend this conclusion to the entire bacterial community. Wild and captive hoopoes breed in nest boxes installed using the same materials, but several environmental differences (including diet) may be the cause of the detected differences. In fact, many bacteria closely related to the OTUs found in hoopoe secretions inhabit the digestive tract of animals [73–76], including birds [77–86]. *Atopobium* has appeared in the faeces of two different passerine species [87,88], whereas *Eubacterium* and *Ruminococcus* occur in chickens and gulls [83,84]. Finally, several species of *Clostridium* have been detected in some of these systems [77,83,84]. Therefore, if bacteria in the uropygial gland originate from the digestive tract of hoopoes, diet differences among captive and wild individuals may explain the detected differences in bacterial communities. Another possible explanation for the detected differences between wild and captive populations is the influence of the migratory behavior of hoopoes. While hoopoes maintained in captivity are kept in a similar environment the whole year, part of the wild hoopoe population breeding in our study area migrates to African winter quarters [89]. Therefore, migrating individuals are exposed to quite different environments from which they may acquire new bacteria. Winter quarter characteristics affect plumage microbiota of migratory birds [90] and, thus, may also explain the higher diversity of the bacterial community of wild hoopoes. All these possibilities deserve further research to untangle the bacterial colonization process of the hoopoe uropygial gland and the possible sources of variation affecting the composition of its symbiotic community (especially in the wild).

## Conclusions

Our results indicate that the bacterial community residing in the uropygial gland of female and nestling hoopoes is much more complex than previously reported. It includes at least 18 species belonging to 16 different genera and 13 families. Composition is largely invariable across individuals, although partially influenced by the environment as suggested by the differences found between wild and captive hoopoes. These results open interesting new avenues of research in the understanding of the relationship between hoopoes and bacteria. Particularly intriguing is the possibility of different bacterial groups functioning additively (e.g. antimicrobial characteristics) or in different contexts (e.g. antipredatory, antiparasitic, or immunological). Moreover, the nature of interactions among the different taxa sharing the gland may be key in the ecological processes determining the final community composition, which can include bacteria acquired from different sources.
